# Immunomodulatory Effects of Wheat Peptides and a Novel Octapeptide (GFNDLGKR) in Immunosuppressed Zebrafish and Mouse Models

**DOI:** 10.1002/fsn3.71948

**Published:** 2026-06-12

**Authors:** Fei Shen, Jiahao Chen, Lina Xiong, Yujie Zhou, Yunjing Han, Juan Du, Wei Zhang, Fengqin Feng, Jiaojiao Zhang, Guanghua He, Lida Wang

**Affiliations:** ^1^ School of Biological and Chemical Engineering Zhejiang University of Science & Technology Hangzhou China; ^2^ College of Biosystems Engineering and Food Science Zhejiang University Hangzhou China; ^3^ Hangzhou Kangyuan Food Science & Technology Co., Ltd. Hangzhou China; ^4^ Beingmate (Hangzhou) Food Research Institute Co., Ltd. Hangzhou China; ^5^ Doppler Ultrasonic Department Zhejiang Provincial Dermatology Hospital Huzhou China; ^6^ Biological Physics Group University of Manchester Manchester UK; ^7^ College of Food and Health Zhejiang A& F University Hangzhou China

**Keywords:** CHRM1, GFNDLGKR, IFN‐γ, immunosuppression, wheat peptides, zebrafish

## Abstract

Immunosuppression increases susceptibility to infections, highlighting the need for safe immunomodulatory strategies. This study investigated the immunomodulatory effects of wheat peptides (WP) and identified bioactive peptide sequences using zebrafish and mouse models. In chloramphenicol (CAP)‐induced immunosuppressed zebrafish, WP (150 μg/mL) significantly restored macrophage counts (32.25% increase), neutrophil fluorescence intensity (28.87% increase), and IFN‐γ levels (42.34% increase). In cyclophosphamide (CTX)‐induced immunosuppressed mice, WP (0.5 g/kg) significantly increased spleen index, thymus index, serum IgA and IFN‐γ levels, and fecal short‐chain fatty acid concentrations. LC–MS/MS peptidomics identified peptide sequences; molecular docking targeting CHRM1 revealed that the octapeptide GFNDLGKR (GR8) exhibited the strongest binding affinity (CDOCKER energy = −161.15 kcal/mol). GR8 (10 μg/mL) validation in zebrafish significantly increased macrophage counts (60.62%), neutrophil fluorescence intensity (44.03%), and IFN‐γ levels (60.84%). These findings demonstrate that WP exerts immunomodulatory effects through restoration of immune cells, organ indices, cytokines, and short‐chain fatty acids (SCFA), and identify GR8 as a promising bioactive peptide for functional food development.

## Graphical abstract

Wheat peptides (WP) exerted immunomodulatory effects in both zebrafish and mouse immunosuppressed models, restoring immune cells, cytokines, organ indices, and SCFAs. A novel octapeptide, GFNDLGKR (GR8), was identified via LC–MS/MS and molecular docking targeting CHRM1. GR8 demonstrated potent immunomodulatory activity in zebrafish, validating its potential as a bioactive peptide for functional food development.

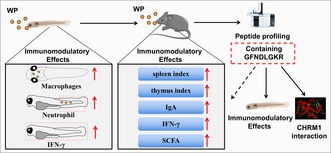

## 1 Introduction

Immunosuppression represents a significant clinical challenge associated with increased susceptibility to infectious diseases, impaired wound healing, and elevated morbidity and mortality rates (Román‐Montes et al. 2026). Immunosuppressed individuals exhibit attenuated inflammatory responses and a broader spectrum of causative pathogens, complicating both diagnosis and clinical management (Alqadi et al. 2025). While pharmacological interventions such as cyclosporine and chemotherapeutic agents like cyclophosphamide (CTX) are effective for their primary indications, they often induce unintended immunosuppression accompanied by significant toxicities, including nephrotoxicity, hepatotoxicity, and gastrointestinal complications (Wu et al. 2018). Consequently, the development of safe and effective immunomodulatory strategies has emerged as a critical research priority.

In recent years, bioactive peptides derived from food proteins have garnered substantial attention as promising immunomodulatory agents due to their favorable safety profiles, low allergenicity, and cost‐effectiveness (Dong and Xue 2025; Zhao et al. 2025). These naturally derived compounds offer distinct advantages over conventional pharmaceuticals, including superior biocompatibility and reduced risk of adverse effects (Cruz‐Chamorro et al. 2020). A growing body of evidence has demonstrated that various food‐derived peptides exert immunomodulatory effects through diverse mechanisms. For example, selenium‐enriched egg white peptides have been shown to effectively counteract cyclophosphamide‐induced reductions in immune organ indices and serum cytokine levels in immunosuppressed mice (Chalamaiah et al. 2018). Soybean peptides facilitate macrophage proliferation and enhance nitric oxide (NO) and cytokine production through toll‐like receptor (TLR) signaling pathways (Wang, Shen, et al. 2022; Wang, Fu, et al. 2022). Furthermore, cereal‐derived peptides have also been reported to possess immunomodulatory properties. Peptides derived from quinoa and rice seeds have been shown to enhance macrophage phagocytic activity and activate systemic immunity (Pomatto et al. 2023). Collectively, these findings underscore the potential of food‐derived bioactive peptides as promising immunomodulatory agents.

Among cereal proteins, wheat gluten represents a particularly attractive source for bioactive peptide production. Our previous studies have demonstrated that wheat peptides (WP) exhibit multiple beneficial activities, including hypoglycemic effects, antioxidant, anti‐inflammatory, and intestinal barrier‐protective effects, and relief from constipation, making them particularly suitable for middle‐aged and elderly populations (Shen et al. 2023, 2026; Wang, Shen, et al. 2022; Wang, Fu, et al. 2022; Wang et al. 2023). Given the reported immunomodulatory effects of various cereal‐derived peptides, we hypothesized that WP may also possess immunomodulatory properties. If validated, such effects would position WP as an ideal nutritional supplement for middle‐aged and elderly individuals, particularly given the additional advantages of wheat as a low‐cost and readily available raw material. Therefore, investigating the immunomodulatory potential of WP holds both scientific and practical significance.

Moreover, WP represent a complex mixture of peptide species with varying chain lengths, molecular weights, and amino acid compositions. The specific peptide sequences responsible for the putative immunomodulatory activity of WP remain largely unknown. Identifying and characterizing these bioactive peptide constituents is essential not only for understanding the molecular mechanisms underlying the immunomodulatory effects but also for establishing quality control standards and enabling targeted production of the most potent immunomodulatory peptides. Therefore, beyond evaluating the overall immunomodulatory efficacy of WP, it is equally important to identify the specific bioactive peptide sequences that contribute to this activity.

In the present study, we aimed to investigate the immunomodulatory effects of WP using complementary zebrafish and mouse immunosuppressed models. Specifically, we established chloramphenicol (CAP)‐induced immunosuppressed zebrafish models to evaluate the effects of WP on macrophage and neutrophil populations, as well as interferon‐gamma (IFN‐γ) and immunoglobulin A (IgA) levels. Additionally, we employed a CTX‐induced immunosuppressed mouse model to assess the effects of WP on immune organ indices, serum cytokine profiles, and fecal short‐chain fatty acid (SCFA) composition. Subsequently, we utilized LC–MS/MS‐based peptidomics combined with molecular docking targeting CHRM1 to identify and characterize bioactive peptide sequences within WP. The lead peptide, GFNDLGKR (designated GR8), was further evaluated for its immunomodulatory activity in the CAP‐induced zebrafish model. Our findings provide mechanistic insights into the immunomodulatory potential of wheat‐derived peptides and identify GR8 as a promising candidate for functional food development.

## 2 Materials and Methods

### 2.1 Materials

Wheat peptides (WP) were prepared by mixing wheat gluten with water at a mass ratio of 1:10. The pH was adjusted to 8.0 ± 0.2 using 1 M NaOH, and the mixture was heated to 50°C under continuous stirring. And alkaline protease (1.0% of gluten weight) was added for 30 min of hydrolysis, during which the pH was maintained at ≥ 7.5 by intermittent addition of 1 M NaOH. Subsequently, neutral protease (1.5% of gluten weight) was added for 60 min, followed by flavor protease (0.5% of gluten weight) for 30 min. The temperature was kept at 50°C throughout the entire enzymatic process. The enzyme was inactivated by heating at 100°C for 30 min. The resulting solution was concentrated to 25°Bx and spray‐dried to obtain WP powder. The molecular weight and amino acid composition are shown in Tables S1 and S2.

CAP was purchased from Tianjin Baima Technology Co. Ltd. (Tianjin, China). CTX and 2‐ethylbutyric acid were purchased from Sigma‐Aldrich (USA). Wild‐type AB and Tg (lyz:DsRed) transgenic zebrafish were obtained from the Zebrafish Platform at Zhejiang University (Hangzhou, China) and maintained in a standardized recirculation system. Enzyme‐linked immunosorbent assay (ELISA) kits for IFN‐γ and IgA were purchased from Wuhan GeneMei Biotechnology Co. Ltd. (Wuhan, China).

### 2.2 Investigation of the Immunomodulatory Effects of WP in a CAP‐Induced Immunosuppressed Zebrafish Model

#### 2.2.1 Effect of WP on Macrophage Proliferation

**FIGURE 1:**
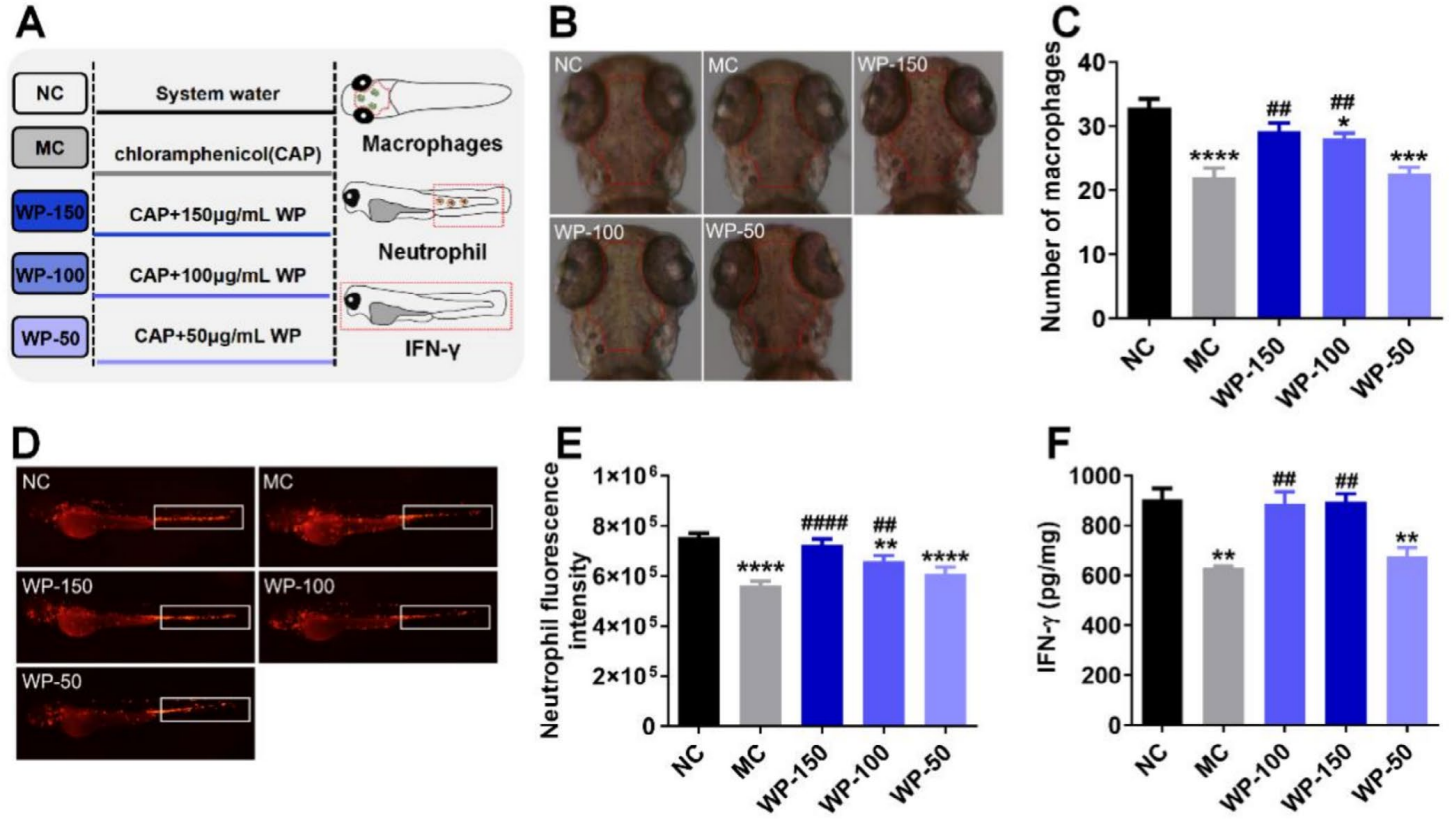
WP improved immunomodulatory effects in a chloramphenicol (CAP)‐induced immunosuppressed zebrafish model (A) Schematic representation of the experimental design. (B) Representative images of head macrophages in AB strain zebrafish following WP treatment. (C) Quantification of macrophage numbers in the head region. (D) Representative fluorescence images of *Tg*(*lyz:DsRed*) transgenic zebrafish showing neutrophil distribution. (E) Quantification of fluorescence intensity in the cloaca‐to‐tail region. (F) IFN‐γ levels in AB strain zebrafish. Data presented as the mean ± SEM. *p < 0.05, **p < 0.01, ***p < 0.001, ****p < 0.0001 versus NC; ^##^ *p* < 0.01, ^####^ *p* < 0.0001 versus MC.

Wild‐type AB zebrafish embryos were obtained from the Zebrafish Platform at Zhejiang University and raised using standard protocols (Approval number ZJU 20220189). The assay for the effect of WP on macrophage proliferation was performed according to methods established in our previous study (Wang et al. 2025). Wild‐type AB zebrafish embryos were treated with 10 μL/mL phenylthiourea (PTU) at 24 h post‐fertilization (hpf). At 48 hpf, dechorionated embryos were distributed into six‐well plates (10 embryos per well, 3 wells per group) and subjected to the following treatments: (1) Negative control (NC): 0.5% DMSO in system water with PTU; (2) Model control (MC): 125 μg/mL CAP (dissolved in DMSO, final concentration 0.5%) in system water with PTU; (3) WP treatment groups: 150, 100, or 50 μg/mL WP combined with 125 μg/mL CAP in system water with PTU (0.5% DMSO). After 24 h, embryos were incubated with 2.5 μg/mL neutral red dye and PTU in the dark for 6 h, washed with system water under dark conditions, anesthetized, fixed in 6% methylcellulose, and imaged under a stereomicroscope. Macrophages in the head region were counted. The experimental design is shown in Figure 1A.

#### 2.2.2 Effect of WP on Neutrophil Proliferation

The method refer to our previous method (Wang et al. 2025). Tg (Lyz:DsRed) transgenic zebrafish larvae were used. At 24 hpf, embryos were treated with 10 μL/mL PTU. At 48 hpf, dechorionated embryos expressing the fluorescent reporter were selected under a fluorescence stereomicroscope and placed into six‐well plates (10 embryos per well, 3 wells per group). Grouping was the same as in section 2.2.1, with CAP at 125 μg/mL. After 24 h, embryos were washed, anesthetized, and imaged under a fluorescence stereomicroscope. Fluorescence intensity from the cloaca to the tip of the tail was quantified.

#### 2.2.3 Effect of WP on IFN‐γ Levels

Refer to our previous method (Wang et al. 2025), wild‐type AB zebrafish embryos were treated with 10 μL/mL PTU at 24 hpf. At 48 hpf, dechorionated embryos were placed into six‐well plates (120 embryos per well, 3 wells per group). Grouping was the same as in sections 2.2.1 and 2.2.2, except that the CAP concentration was increased to 150 μg/mL based on preliminary dose–response optimization (125 μg/mL was sufficient for innate immune endpoints, while 150 μg/mL was required for consistent IFN‐γ suppression). After 24 h of incubation at 28.5°C, embryos were collected, washed with PBS, and homogenized in PBS (50 μL per 10 mg tissue) using a tissue grinder (90 s). The homogenate was centrifuged (4°C, 5000 rpm, 5 min), and the supernatant was used to measure IFN‐γ levels using an ELISA kit according to the manufacturer's instructions.

### 2.3 Investigation of the Immunomodulatory Effects of WP in a CTX‐Induced Immunosuppressed Mouse Model

#### 2.3.1 Animals and Housing

Forty‐five male specific‐pathogen‐free (SPF) ICR mice (6–11 weeks old, body weight 35 ± 2 g) were purchased from Shanghai SLAC Laboratory Animal Co. Ltd. via Zhejiang Chinese Medical University. The animal experiment was approved by the Institutional Animal Care and Use Committee (approval No. IACUC‐20230206‐07). Mice were housed under controlled conditions (22°C ± 2°C, 50% ± 10% relative humidity, 12 h light/dark cycle).

#### 2.3.2 Experimental Design and Treatment

**FIGURE 2:**
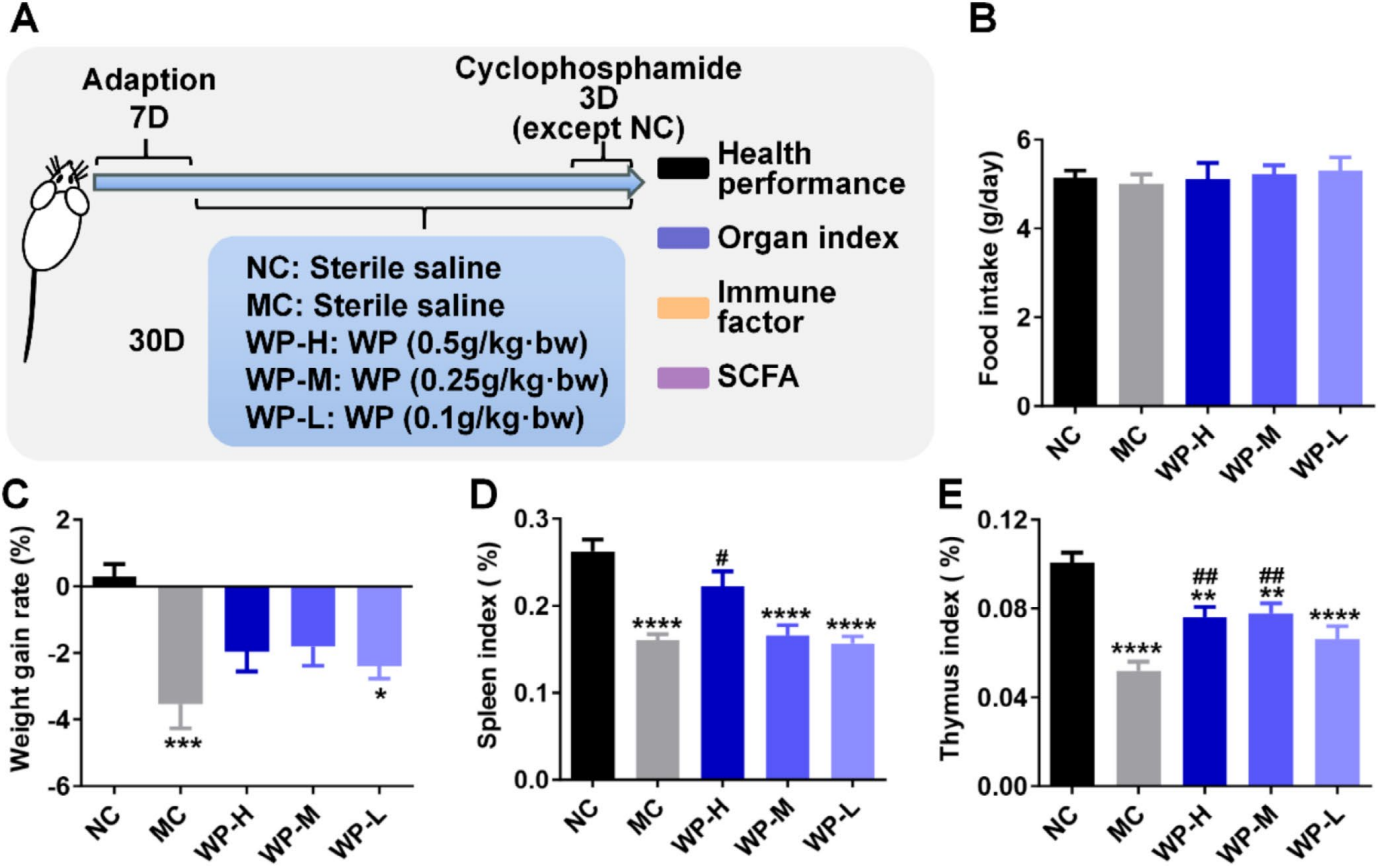
WP supplementation increased immune organ indices in cyclophosphamide (CTX)‐induced immunosuppressed mouse model. (A) Design of the mouse experiment, (B) food intake, (C) weight gain rate, (D) spleen index, (E) thymus index. Data presented as the mean ± SEM. *p < 0.05, **p < 0.01, ***p < 0.001, ****p < 0.0001 versus NC; ^#^ *p* < 0.05, ^##^ *p* < 0.01 versus MC.

The experimental design is shown in Figure 2A. Mice were randomly divided into five groups (n=9 per group): normal control (NC), model control (MC), and three WP treatment groups (high dose: 0.5 g/kg, WP‐H; medium dose: 0.25 g/kg, WP‐M; low dose: 0.1 g/kg, WP‐L). WP was administered daily by gavage for 30 consecutive days at a volume of 10 μL per gram body weight (10 μL/g). The gavage volume was adjusted individually based on body weight measured before each administration to ensure dose accuracy. The NC and MC groups received an equal volume of saline. From day 28 to day 30, the MC and WP‐treated groups were intraperitoneally injected with CTX (50 mg/kg/day) to induce immunosuppression, whereas the NC group received saline. Body weight and food intake were recorded before and after CTX administration.

#### 2.3.3 Immune Organ Index

The spleen and thymus were collected, washed in pre‐chilled phosphate‐buffered saline (PBS, 0.01 mol/L, pH 7.4), and blotted dry. Organ indices were calculated as follows: organ weight (g)/body weight (g).

#### 2.3.4 Serum Cytokine and IgA Levels

Serum cytokine and IgA levels were measured using the ELISA method, referring to our previous protocol (Shen et al. 2023, 2026). Serum levels of IFN‐γ and IgA were measured using commercial ELISA kits (GeneMei Biotechnology Co. Ltd., Wuhan, China) according to the manufacturer's instructions.

#### 2.3.5 Fecal Short‐Chain Fatty Acid (SCFA) Analysis

For the method of determining SCFA, refer to our previous method (Shen et al. 2023). Fecal concentrations of acetic acid, propionic acid, and butyric acid were determined. The standards for acetic acid (≥ 99.8%), propionic acid (≥ 99.5%), and butyric acid (≥ 99.5%) were purchased from Sigma‐Aldrich (St. Louis, MO, USA). Calibration curves were prepared using serial dilutions, and all samples were analyzed in triplicate. Approximately 50 mg of feces was mixed with 250 μL of sterile ultrapure water, vortexed for 5 min, and then adjusted to pH 2–3 with 10 μL of 5 mol/L HCl. After vortexing for 1 min and resting at room temperature for 5 min, the mixture was centrifuged at 12,000 rpm for 30 min at 4°C. A 200 μL aliquot of the supernatant was mixed with 0.5 μL of 20‐fold diluted 2‐ethylbutyric acid (internal standard), vortexed for 1 min, and centrifuged at 12,000 rpm for 5 min at 4°C. The supernatant (150 μL) was used for gas chromatography (GC) analysis. GC was performed using an Agilent DB‐FFAP125‐3237 column (30 m × 0.52 mm × 0.50 mm). The temperature program was as follows: initial temperature 100°C held for 0.5 min, increased to 180°C at 8°C/min and held for 1 min, and finally increased to 240°C at 20°C/min and held for 15 min. The injector and flame ionization detector temperatures were 200°C and 240°C, respectively. Hydrogen, air, and nitrogen flow rates were 30, 300, and 20 mL/min, respectively. The injection volume was 1 μL.

### 2.4 Screening of Bioactive Peptides

WP were subjected to LC–MS/MS analysis for peptide identification. The resulting peptide sequences were filtered based on predefined criteria and further evaluated by molecular docking to identify the most promising bioactive peptides based on docking scores. This method is based on our previous approach (Wang et al. 2025).

#### 2.4.1 Identification of Wheat Peptide Sequences

WP samples were dissolved in NH₄HCO₃ solution, reduced with dithiothreitol at 56°C for 1 h, and alkylated with iodoacetamide in the dark for 40 min. After desalting and solvent evaporation, the samples were redissolved in 10 μL of mobile phase A (0.1% formic acid) for LC–MS/MS analysis. Chromatographic separation was performed on an Acclaim PepMap RPLC C18 analytical column (150 μm × 150 mm, 3 μm) at a flow rate of 600 nL/min. Mobile phase A was 0.1% formic acid, and mobile phase B was 0.1% formic acid in 80% acetonitrile. The gradient program was: 0–2 min, 4%–8% B; 2–45 min, 8%–40% B; 45–55 min, 40%–60% B; 55–56 min, 60%–95% B; 56–66 min, 95% B. Mass spectrometry was performed using an Orbitrap instrument. Full MS scans were acquired in the range of 100–1500 m/z at a resolution of 70,000, with a maximum injection time of 100 ms and an automatic gain control (AGC) target of 3 × 10^6^. The top 20 precursor ions were selected for MS/MS fragmentation using high‐energy collisional dissociation (HCD), and MS/MS spectra were acquired at a resolution of 17,500, with a maximum injection time of 50 ms and an AGC target of 1 × 10^5^. Raw data were processed using PEAKS Studio with de novo sequencing.

#### 2.4.2 Screening of Peptides With Potential Immunomodulatory Activity

The acetylcholine receptor is involved in immune regulation, as its activation suppresses inflammation and enhances immune cell function. Therefore, identified peptides were subjected to molecular docking with the M_1_ muscarinic acetylcholine receptor (CHRM1). Peptides were selected based on the following criteria: average local confidence (ALC) > 95%, peak area > 2 × 10^6^, and PeptideRanker score > 0.8. The crystal structure of CHRM1 (PDB ID: 5CXV) was obtained from the Protein Data Bank. Using Discovery Studio, water molecules were removed, hydrogen atoms were added, and the active site was defined. The three‐dimensional structures of the selected peptides were constructed, and their energies were minimized using the CHARMm force field. CDOCKER was used to simulate binding modes, binding sites, and interacting residues with the lowest binding energy and highest affinity. Candidate peptides were prioritized based on binding energy and hydrogen bond count.

### 2.5 Investigation of the Immunomodulatory Effects of GR8 in a CAP‐Induced Immunosuppressed Zebrafish Model

#### 2.5.1 Effect of GR8 on Macrophage Proliferation

Wild‐type AB zebrafish embryos were treated with 10 μL/mL PTU at 24 hpf. At 48 hpf, dechorionated embryos were distributed into six‐well plates (10 embryos per well, 3 wells per group). The groups were: (1) Negative control (NC): 0.5% DMSO in system water with PTU; (2) Model control (MC): 125 μg/mL CAP (0.5% DMSO) in system water with PTU; (3) Wheat peptide control (WP‐10): 10 μg/mL WP plus 125 μg/mL CAP in system water with PTU (0.5% DMSO); (4) Octapeptide treatment groups (GR8‐10, GR8‐5, GR8‐1, GR8‐0.1): 10, 5, 1, or 0.1 μg/mL GFNDLGKR octapeptide (GR8) combined with 125 μg/mL CAP in system water with PTU (0.5% DMSO). After 24 h, embryos were incubated with 2.5 μg/mL neutral red dye and PTU in the dark for 6 h, washed, anesthetized, fixed in 6% methylcellulose, and imaged under a stereomicroscope. Macrophages in the head region were counted. The experimental design is shown in Figure 6A.

#### 2.5.2 Effect of GR8 on Neutrophil Proliferation

Tg (Lyz:DsRed) zebrafish larvae were treated with 10 μL/mL PTU at 24 hpf. At 48 hpf, dechorionated embryos expressing the fluorescent reporter were selected and placed into six‐well plates (10 embryos per well, 3 wells per group). Grouping was identical to section 2.5.1, with CAP at 125 μg/mL. After 24 h, embryos were washed, anesthetized, and imaged under a fluorescence stereomicroscope. Fluorescence intensity from the cloaca to the tip of the tail was quantified.

#### 2.5.3 Effect of GR8 on IFN‐γ Levels

Wild‐type AB zebrafish embryos were treated with 10 μL/mL PTU at 24 hpf. At 48 hpf, dechorionated embryos were placed into six‐well plates (120 embryos per well, 3 wells per group). Grouping was the same as in sections 2.5.1 and 2.5.2, except that the CAP concentration was increased to 150 μg/mL. After 24 h at 28.5°C, embryos were collected, washed with PBS, and homogenized in PBS (50 μL per 10 mg tissue) using a tissue grinder (90 s). The homogenate was centrifuged (4°C, 5000 rpm, 5 min), and the supernatant was used to measure IFN‐γ level by ELISA.

### 2.6 Data Processing and Statistical Analysis

Statistical analyses were performed using GraphPad Prism version 9.0. Data are presented as mean ± SD or mean ± SEM as indicated in the figure legends. One‐way analysis of variance (ANOVA) followed by Duncan's test was used to determine statistical significance. Differences were considered significant at *p* < 0.05. Graphical representations were generated using the same software.

## 3 Results

### 3.1 Characterization of WP

The molecular weight distribution and amino acid composition of the WP used in this study were characterized prior to the immunomodulatory experiments. As shown in Table S1, the majority of WP (62.71% ± 3.79%) fell within the molecular weight range of 180–3000 Da, indicating that the enzymatic hydrolysis process effectively degraded wheat gluten into small peptides. Additionally, the amino acid composition analysis (Table S2) revealed that glutamine (Gln, 22.91 g/100 g peptide) and proline (Pro, 9.92 g/100 g peptide) were the most abundant amino acids in WP.

### 3.2 WP Improved Immunomodulatory Effects in a CAP‐Induced Immunosuppressed Zebrafish Model

To investigate the immunomodulatory effects of wheat peptides (WP), a CAP‐induced immunosuppressed zebrafish model was established. CAP, a broad‐spectrum antibiotic, has been shown to cause immunotoxicity in zebrafish (Wang et al. 2025). Macrophages and neutrophils are critical components of the innate immune system, responsible for combating infections (Kotlyarov 2022). IFN‐γ is a key cytokine secreted by immune cells that plays an important role in immune regulation (Wu et al. 2025). Compared with the negative control (NC) group, the model control (MC) group exhibited a significant decrease in macrophage count (*p* < 0.0001, Figure 1B,C), neutrophil count (*p* < 0.0001, Figure 1E,F), and IFN‐γ level (*p* < 0.01, Figure 1F), confirming successful establishment of the immunosuppressed model. Compared with the MC group, WP treatment increased macrophage counts by 32.25% (WP‐150, *p* < 0.01), 26.81% (WP‐100, *p* < 0.01), and 2.66% (WP‐50, *p* > 0.05); increased neutrophil fluorescence intensity by 28.87% (WP‐150, *p* < 0.0001), 16.99% (WP‐100, *p* < 0.01) and 8.36% (WP‐50, *p* > 0.05); and increased IFN‐γ levels by 42.34% (WP‐150, *p* < 0.01), 41.18% (WP‐100, *p* < 0.01), and 7.47% (WP‐50, *p* > 0.05). Collectively, these results indicate that WP possesses immunomodulatory potential.

### 3.3 WP Supplementation Increased Immune Organ Indices in CTX‐Induced Immunosuppressed Mouse Model

To further evaluate the immunomodulatory effects of WP, a CTX‐induced immunosuppressed mouse model was used. CTX, a commonly used chemotherapeutic agent, induces systemic immunosuppression characterized by intestinal barrier disruption and impaired IgA‐mediated immune responses (Ren et al. 2026). No significant difference in food intake was observed between the CTX‐treated group and the NC group (*p* > 0.05, Figure 2B). Compared with the NC group, the MC group showed significantly reduced body weight (*p* < 0.001, Figure 2C) and decreased spleen and thymus indices (*p* < 0.0001, Figure 2D,E), confirming successful model establishment. At the highest dose (WP‐H, 0.5 g/kg), body weight and spleen index were not significantly different from those of the NC group (*p* > 0.05, Figure 2C,D). Compared with the MC group, WP‐H significantly increased both spleen index (*p* < 0.05, Figure 2D) and thymus index (*p* < 0.01, Figure 2E). At the medium dose (WP‐M, 0.25 g/kg), body weight showed no significant difference from the NC group (*p* > 0.05, Figure 2C), while thymus index was significantly increased compared with the MC group (*p* < 0.01, Figure 2E). At the lowest dose (WP‐L, 0.1 g/kg), body weight was significantly lower than that of the NC group (*p* < 0.05, Figure 2C), though the magnitude of reduction was less pronounced than that observed between MC and NC groups (*p* < 0.001, Figure 2C). These results suggest that WP exerts dose‐dependent immunomodulatory effects within the range of 0.1–0.5 g/kg.

### 3.4 WP Supplementation Increased IgA and IFN‐γ Levels in CTX‐Induced Immunosuppressed Mouse Model

**FIGURE 3:**
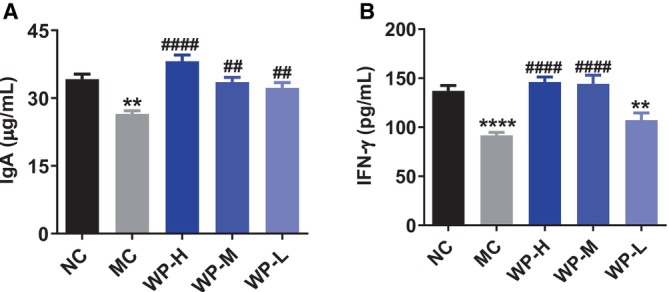
WP supplementation increased IgA and IFN‐γ levels in a CTX‐induced immunosuppressed mouse model. (A) serum IgA content, (B) serum IFN‐γ content. Data presented as the mean ± SEM. **p < 0.01, ****p < 0.0001 versus NC; ^##^ *p* < 0.01, ^####^ *p* < 0.0001 versus MC.

The effects of WP on serum IgA and IFN‐γ levels are shown in Figure 3. CTX‐induced immunosuppression resulted in significantly reduced levels of both IFN‐γ (*p* < 0.01, Figure 3A) and IgA (*p* < 0.0001, Figure 3B) compared with the NC group. Compared with the MC group, WP‐H and WP‐M significantly upregulated both IgA and IFN‐γ levels (*p* < 0.01 to *p* < 0.0001, Figure 3A,B). WP‐L significantly increased IgA levels compared with the MC group (*p* < 0.01, Figure 3A) but did not significantly affect IFN‐γ levels.

### 3.5 WP Supplementation Modulated Short‐Chain Fatty Acids (SCFA) in CTX‐Induced Immunosuppressed Mouse Model

**FIGURE 4:**
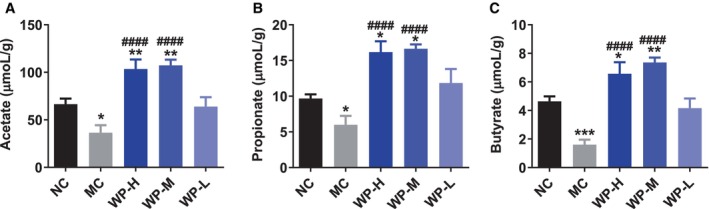
WP supplementation modulated short‐chain fatty acids (SCFA) in CTX‐induced immunosuppressed mouse model. Fecal (A) acetate content, (B) propionate content, (C) butyrate content. Data presented as the mean ± SEM. *p < 0.05, **p < 0.01, ***p < 0.001 versus NC; ^####^ *p* < 0.0001 versus MC.

SCFA regulate nutrient absorption, hormone production, energy metabolism, and intestinal barrier repair, and their composition is closely associated with intestinal mucosal immunity (Qian et al. 2026). The effects of WP on fecal SCFA levels are shown in Figure 4. Compared with the NC group, the MC group exhibited significantly reduced levels of acetic acid, propionic acid, and butyric acid (*p* < 0.05 to *p* < 0.01, Figure 4A–C). Compared with the MC group, WP‐H and WP‐M significantly increased all three SCFA levels (*p* < 0.0001, Figure 4A–C), indicating that WP at doses of 0.25–0.5 g/kg promotes SCFA recovery.

### 3.6 Identification of Potential CHRM1‐Binding Peptides

**FIGURE 5:**
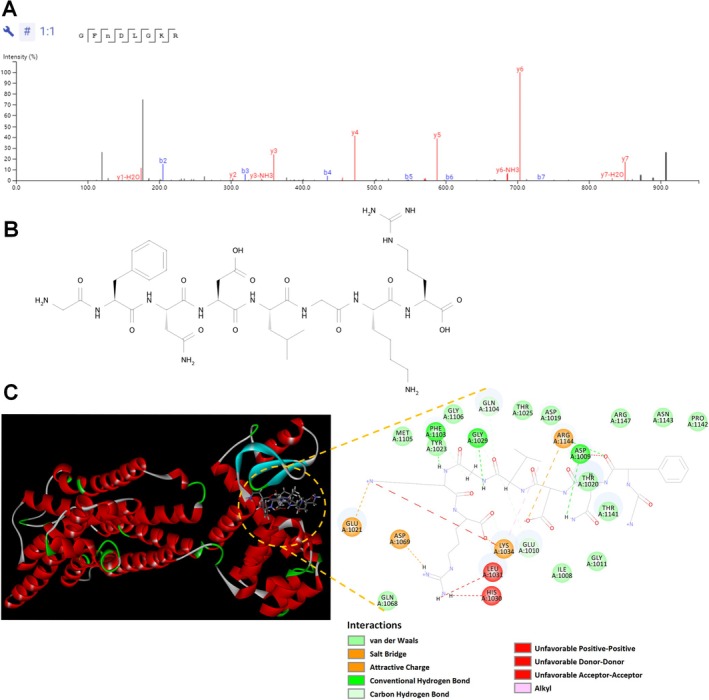
Molecular docking between GR8 and CHRM1. (A) Secondary ion mass spectrometry of GR8, (B) Chemical structural formula of GR8, (C) Molecular interaction of GR8 with CHRM1.

LC–MS/MS analysis identified peptide sequences from WP with average local confidence scores exceeding 95%. Applying screening criteria (PeptideRanker score > 0.8; relative intensity > 1 × 10^6^), potentially bioactive peptides were selected for molecular docking with the M_1_ muscarinic acetylcholine receptor (CHRM1). Of these, peptides exhibited binding to CHRM1. Among them, GFNDLGKR (GR8) demonstrated the strongest binding affinity, with a CDOCKER energy of −161.15 kcal/mol, suggesting its potential immunomodulatory efficacy. Figure 5A,B present the secondary ion mass spectrometry confirmation and the chemical structural formula of GR8, respectively. To further elucidate its binding mode, the molecular interaction between GR8 and CHRM1 was analyzed, as shown in Figure 5C. Analysis of the molecular interactions between GR8 and CHRM1 revealed that binding was mediated primarily by van der Waals forces, hydrogen bonds (including conventional and carbon–hydrogen bonds), hydrophobic interactions (alkyl), and electrostatic interactions (including salt bridges and attractive charge interactions). Specifically, GR8 formed van der Waals interactions with 13 residues (GLN1068, ILE1008, GLY1011, THR1141, THR1020, MET1105, TYR1023, GLY1106, THR1025, ASP1019, ARG1147, ASN1143, and PRO1142); hydrogen bonds with 7 residues (PHE1103, GLY1029, ASP1009, ARG1144, GLU1010, LYS1034, and GLN1104); hydrophobic interactions with LEU1031; and electrostatic interactions with 4 residues (GLU1021, ASP1069, LYS1034, and ARG1144). These results indicate the potential of GR8 to enhance immune function.

### 3.7 GR8 Improved Immunomodulatory Effects in a CAP‐Induced Immunosuppressed Zebrafish Model

**FIGURE 6:**
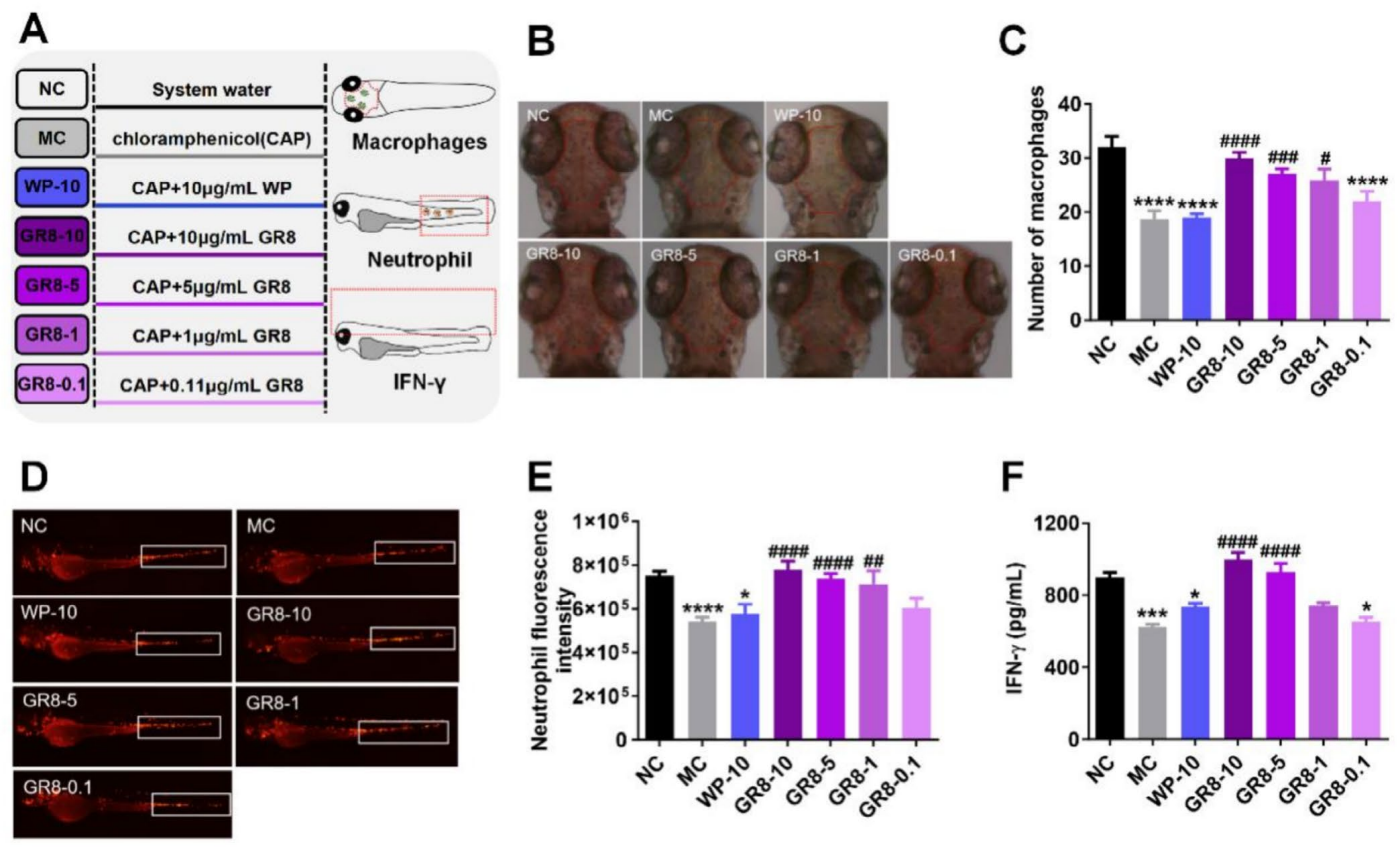
GR8 improved immunomodulatory effects in CAP‐induced immunosuppressed zebrafish model (A) Schematic representation of the experimental design. (B) Representative images of head macrophages in AB strain zebrafish following GR8 treatment. (C) Quantification of macrophage numbers in the head region. (D) Representative fluorescence images of *Tg* (*lyz:DsRed*) transgenic zebrafish showing neutrophil distribution. (E) Quantification of fluorescence intensity in the cloaca‐to‐tail region. (F) IFN‐γ levels in AB strain zebrafish. Data presented as the mean ± SEM. *p < 0.05, ****p < 0.0001 versus NC; ^##^ *p* < 0.01, ^###^ *p* < 0.001, ^####^ *p* < 0.0001 versus MC.

To evaluate the immunomodulatory effects of GR8, a CAP‐induced immunosuppressed zebrafish model was used. The effects of GR8 are shown in Figure 6. Compared with the MC group, the number of macrophages in the treatment groups was increased by 1.95% (WP‐10, *p* > 0.05), 60.62% (GR8‐10, *p* < 0.0001), 45.01% (GR8‐5, *p* < 0.001), 39.19% (GR8‐1, *p* < 0.05), 18.28% (GR8‐0.1, *p* > 0.05); The neutrophil fluorescence intensity in the treatment groups was increased by 7.01% (WP‐10, *p* > 0.05), 44.03% (GR8‐10, *p* < 0.0001), 36.38% (GR8‐5, *p* < 0.0001), 31.88% (GR8‐1, *p* < 0.01), 11.70% (GR8‐0.1, *p* > 0.05); The IFN‐γ in the treatment groups was increased by 18.91% (WP‐10, *p* > 0.05), 60.84% (GR8‐10, *p* < 0.0001), 49.94% (GR8‐5, *p* < 0.0001), 19.26% (GR8‐1, *p* > 0.05), 5.28% (GR8‐0.1, *p* > 0.05). Collectively, these findings demonstrate that GR8 administration significantly improves immune function in immunosuppressed zebrafish.

## 4 Discussion

In the present study, we systematically investigated the immunomodulatory effects of wheat peptides (WP) using complementary zebrafish and mouse immunosuppressed models, and further identified a novel bioactive octapeptide, GFNDLGKR (GR8), through LC–MS/MS‐based peptidomics combined with molecular docking targeting CHRM1. Our results demonstrated that WP significantly restored macrophage and neutrophil populations, as well as IFN‐γ levels, in CAP‐induced immunosuppressed zebrafish. In a CTX‐induced immunosuppressed mouse model, WP supplementation effectively increased immune organ indices, serum IgA and IFN‐γ levels, and fecal SCFA concentrations. Moreover, the identified peptide GR8 exhibited potent immunomodulatory activity in the CAP‐induced zebrafish model, validating its role as a key bioactive component within WP. These findings collectively establish WP as a promising immunomodulatory agent and identify GR8 as a lead peptide for functional food development.

The zebrafish model employed in this study provided a rapid and high‐throughput platform for assessing innate immune responses. CAP, a broad‐spectrum antibiotic, has been demonstrated to induce immunotoxicity in zebrafish, characterized by reduced populations of macrophages and neutrophils, which are critical components of the innate immune system (Bai and Tang 2023). In our study, CAP exposure significantly decreased macrophage counts, neutrophil fluorescence intensity, and IFN‐γ levels, confirming successful model establishment. WP treatment at concentrations of 150 and 100 μg/mL significantly reversed these immunosuppressive effects, restoring immune cell populations and cytokine levels to partially restored values. These observations are consistent with previous reports demonstrating that food‐derived peptides, such as selenium‐enriched egg white peptides and soybean peptides, can enhance innate immune responses in immunosuppressed models (Miltenburg et al. 2026). Notably, the dose‐dependent response observed in our study, with WP‐150 exhibiting the most pronounced effects, suggests that the immunomodulatory activity of WP is concentration‐dependent.

To further evaluate the systemic immunomodulatory effects of WP, we employed a CTX‐induced immunosuppressed mouse model, which is widely used to assess both innate and adaptive immune responses (Cong et al. 2026). Here, CTX treatment resulted in significant reductions in body weight, spleen and thymus indices, and serum IgA and IFN‐γ levels, all of which are characteristic features of immunosuppression. WP administration, particularly at the high (0.5 g/kg) and medium (0.25 g/kg) doses, effectively reversed these changes. The restoration of spleen and thymus indices is particularly noteworthy, as these organs play central roles in lymphocyte development and immune response initiation (Liu and Huang 2025). The observed increase in serum IgA levels suggests that WP may enhance humoral immunity, while the elevation of IFN‐γ indicates a potential role in promoting Th1‐type cellular immune responses. These findings align with previous studies showing that bioactive peptides can modulate both humoral and cellular immune responses (Chambers et al. 2024).

An important finding of our study is the effect of WP on fecal SCFA profiles. SCFA, particularly acetate, propionate, and butyrate, are fermentation products of dietary fiber by gut microbiota and play crucial roles in maintaining intestinal barrier integrity and modulating mucosal immunity (Zhang, Fan, et al. 2026a). Butyrate, in particular, has been shown to enhance the differentiation of regulatory T cells and promote IgA production (Tang et al. 2022). In our study, CTX treatment significantly reduced all three SCFA levels, consistent with the known effects of CTX on gut microbiota composition (He et al. 2026). WP‐H and WP‐M administration significantly restored SCFA levels, suggesting that WP may exert immunomodulatory effects, at least in part, through modulation of gut microbiota and SCFA production. This observation is consistent with recent reports demonstrating that dietary peptides can influence gut microbiota composition and SCFA profiles (Costa et al. 2023). Given the established link between SCFA and immune function, this finding provides a plausible mechanistic basis for the systemic immunomodulatory effects of WP.

To elucidate the molecular basis of WP's immunomodulatory activity, we employed LC–MS/MS‐based peptidomics to identify the peptide sequences within WP. Among the identified peptides, selected based on stringent criteria (ALC > 95%, peak area > 2 × 10^6^, PeptideRanker score > 0.8) and subjected to molecular docking with CHRM1. CHRM1 was selected as a target because emerging evidence has implicated muscarinic acetylcholine receptors in immune regulation. Specifically, activation of CHRM1 on immune cells has been shown to modulate inflammatory responses and enhance immune cell function (Yan et al. 2022). In our docking analysis, GFNDLGKR (GR8) demonstrated the strongest binding affinity (CDOCKER energy = −161.15 kcal/mol). The extensive interactions between GR8 and CHRM1, including van der Waals forces, hydrogen bonds, hydrophobic interactions, and electrostatic interactions, suggest a stable and specific binding mode. These in silico findings indicate that GR8 may exert its immunomodulatory effects through CHRM1‐mediated signaling pathways.

The immunomodulatory activity of GR8 was subsequently validated in the CAP‐induced immunosuppressed zebrafish model. GR8 treatment at concentrations of 10, 5, and 1 μg/mL significantly increased macrophage counts, neutrophil fluorescence intensity, and IFN‐γ levels, with the 10 μg/mL dose exhibiting the most pronounced effects. Notably, the effects of GR8‐10 were superior to those of WP‐10 (10 μg/mL WP), suggesting that GR8 is a key bioactive component contributing to the overall immunomodulatory activity of WP. The dose‐dependent response observed further supports the specificity of GR8's effects. These findings are consistent with previous reports demonstrating that specific peptide sequences isolated from protein hydrolysates can exhibit potent biological activities that surpass those of the parent hydrolysate (Shen et al. 2025).

Other cereal‐derived peptides have also been reported to possess immunomodulatory properties. For example, the rice albumin‐derived octapeptide oryzatensin (Gly‐Tyr‐Pro‐Met‐Tyr‐Pro‐Leu‐Pro‐Arg) enhances phagocytosis of human polymorphonuclear leukocytes and superoxide anion production (Takahashi et al. 1994). Similarly, selenium‐containing peptides from selenium‐enriched rice protein hydrolysates, such as TSeMMM and SeMDPGQQ, activate RAW264.7 macrophages by promoting NO production and phagocytosis (Fang et al. 2019). However, most of these studies have primarily relied on in vitro cell‐based assays or mouse models, with limited exploration of alternative in vivo models. In contrast, milk‐derived and animal‐derived immunomodulatory peptides have been far more extensively characterized. Recent studies have identified and validated numerous peptides with potent immunomodulatory activity from both terrestrial and aquatic animal proteins. For instance, the nonapeptide KEMPFPK derived from bovine β‐casein not only restores immune organ indices and modulates serum cytokine levels but also reshapes the gut microbiota in cyclophosphamide‐induced immunosuppressed mice (Zhang, Zhang, et al. 2026b). Furthermore, protein hydrolysates or specific peptide sequences from fish (e.g., yellowfin tuna, monkfish, Nile tilapia), shellfish (e.g., oysters), and poultry eggs (e.g., egg yolk) have been shown to promote macrophage and dendritic cell activation, stimulate splenocyte and natural killer (NK) cell proliferation, enhance phagocytosis, and increase the expression of cytokines and immunoglobulins, primarily through the activation of NF‐κB and MAPK signaling pathways (Omeje et al. 2026). These findings indicate that the immunomodulatory mechanisms of animal‐derived peptides are relatively well understood, with considerable progress having been made from cellular models to mouse models and even clinical trials. Taken together, while cereal‐derived peptides have demonstrated promising immunomodulatory potential (Ashaolu 2024), the overall body of research remains limited—particularly regarding the transition from cell‐based assays to comprehensive in vivo validation. In our study, the wheat‐derived peptide GR8 restored both innate (macrophages and neutrophils) and adaptive (IFN‐γ) immune parameters simultaneously in a zebrafish model, providing a valuable addition to this underexplored area. Moreover, wheat is globally cultivated, low in cost, and widely available, offering distinct economic and scalability advantages over quinoa or rice‐derived peptides for large‐scale functional food applications.

While several limitations of this study should be acknowledged. First, while we demonstrated that GR8 binds to CHRM1 in silico, direct experimental validation of this interaction, such as through surface plasmon resonance or cellular thermal shift assays, is needed to confirm the molecular mechanism. Second, the precise signaling pathways downstream of CHRM1 activation by GR8 remain to be elucidated. Future studies employing CHRM1 antagonists or CHRM1‐knockdown models would help establish the causal role of this receptor in GR8‐mediated immunomodulation. Third, although we observed WP‐induced restoration of SCFA levels, the causal relationship between SCFA modulation and immune restoration requires further investigation, including gut microbiota profiling and fecal microbiota transplantation experiments. Fourth, regarding the animal models used, the CTX‐induced immunosuppression model primarily recapitulates chemotherapy‐related immunosuppression. Whether WP and GR8 exert similar protective effects against other types of immunosuppression, such as age‐related immunosenescence or immunosuppression associated with autoimmune diseases, remains unknown and warrants further investigation. Finally, the translational potential of WP and GR8 should be evaluated in clinical studies to confirm their efficacy and safety in humans.

In conclusion, this study demonstrates that WP exert significant immunomodulatory effects in both zebrafish and mouse immunosuppressed models, as evidenced by restoration of immune cell populations, immune organ indices, cytokine levels, and SCFA profiles. Through LC–MS/MS‐based peptidomics and molecular docking, we identified GFNDLGKR (GR8) as a novel bioactive octapeptide that binds to CHRM1 and exhibits potent immunomodulatory activity. These findings not only provide mechanistic insights into the immunomodulatory potential of wheat‐derived peptides but also identify GR8 as a promising candidate for functional food development aimed at enhancing immune function in immunocompromised populations. Given the low cost and wide availability of wheat as a raw material, WP and its derived bioactive peptide GR8 represent attractive and accessible options for nutritional intervention.

## Author Contributions

**Wei Zhang:** conceptualization. **Lida Wang:** conceptualization, supervision, project administration, funding acquisition. **Lina Xiong:** conceptualization. **Fengqin Feng:** conceptualization. **Juan Du:** resources. **Jiaojiao Zhang:** investigation, resources. **Fei Shen:** conceptualization, methodology, investigation, formal analysis, writing – original draft, funding acquisition. **Yujie Zhou:** investigation. **Guanghua He:** conceptualization, funding acquisition, supervision. **Jiahao Chen:** investigation, writing – original draft. **Yunjing Han:** investigation.

## Funding

This work was supported by the National Natural Science Foundation of China (No. 32502183), Zhejiang Province “Leading Geese” R&D Projects (2025C04040), Science and Technology Development Plan Project of Hangzhou City (20241203A02), and Zhejiang Provincial Program for Disease Prevention and Control Science and Technology (2026JKY135).

## Conflicts of Interest

The authors declare no conflicts of interest.

## Supporting information


**Table S1:** The molecular weight distribution of WP.
**Table S2:** The amino acid composition of WP.

## Data Availability

The data that support the findings of this study are available on request from the corresponding author. The data are not publicly available due to privacy or ethical restrictions.
