# Supplementary material for: Immunomodulatory Effects of Wheat Peptides and a Novel Octapeptide (GFNDLGKR) in Immunosuppressed Zebrafish and Mouse Models
Source: Food Sci Nutr. 2026 Jun 12;14(6):e71948. doi: 10.1002/fsn3.71948 (PMC13263540; doi:10.1002/fsn3.71948)
Supplement: Supplementary file 1 — Table S1: The molecular weight distribution of WP. Table S2: The amino acid composition of WP. [file FSN3-14-e71948-s001.docx]

**Supplement**

**Table S1.** The molecular weight distribution of WP.

| Molecular weight range (Da) | > 10000 | 5000-10000 | 3000-5000 | 180-3000 | < 180 |
| --- | --- | --- | --- | --- | --- |
| Content (%) | 13.35±2.31 | 11.46±1.92 | 4.63±0.87 | 62.71±3.79 | 7.85±0.64 |

**Table S2.** The amino acid composition of WP.

| Amino acid | Content (g/100g peptide) | Amino acid | Content (g/100g peptide) |
| --- | --- | --- | --- |
| Asp | 1.76 | Tyr | 3.46 |
| Glu | 4.61 | Val | 3.98 |
| Gln | 22.91 | Met | 1.46 |
| Ser | 2.55 | Cys | 0.39 |
| Gly | 2.83 | Ile | 3.81 |
| His | 1.00 | Leu | 5.43 |
| Arg | 3.36 | Phe | 4.25 |
| Thr | 1.59 | Lys | 1.12 |
| Ala | 2.23 | Total | 76.66 |
| Pro | 9.92 |  |  |
